# Crystal structure of the photosensory module from a PAS-less cyanobacterial phytochrome as Pr shows a mix of dark-adapted and photoactivated features

**DOI:** 10.1016/j.jbc.2024.107369

**Published:** 2024-05-14

**Authors:** E. Sethe Burgie, Alayna J. Mickles, Fang Luo, Mitchell D. Miller, Richard D. Vierstra

**Affiliations:** 1 Department of Biology, Washington University in St Louis, St Louis, Missouri, USA; 2 Department of Biosciences, Rice University, Houston, Texas, USA

**Keywords:** phytochrome, cyanobacteria, *Synechococcus sp*. JA-2-3B'a(2–13), PAS-less Phy, X-ray crystallography, red light-absorbing Pr state, bilin chromophore

## Abstract

Phytochromes (Phys) are a diverse collection of photoreceptors that regulate numerous physiological and developmental processes in microorganisms and plants through photointerconversion between red-light-absorbing Pr and far-red light-absorbing Pfr states. Light is detected by an N-terminal photo-sensing module (PSM) sequentially comprised of Period/ARNT/Sim (PAS), cGMP-phosphodiesterase/adenylyl cyclase/FhlA (GAF), and Phy-specific (PHY) domains, with the bilin chromophore covalently-bound within the GAF domain. Phys sense light *via* the Pr/Pfr ratio measured by the light-induced rotation of the bilin D-pyrrole ring that triggers conformational changes within the PSM, which for microbial Phys reaches into an output region. A key step is a β-stranded to α-helical reconfiguration of a hairpin loop extending from the PHY domain to contact the GAF domain. Besides canonical Phys, cyanobacteria express several variants, including a PAS-less subfamily that harbors just the GAF and PHY domains for light detection. Prior 2D-NMR studies of a model PAS-less Phy from *Synechococcus_sp*._JA-2-3B'a(2–13) (*SyB*-Cph1) proposed a unique photoconversion mechanism involving an A-pyrrole ring rotation while magic-angle-spinning NMR probing the chromophore proposed the prototypic D-ring flip. To help solve this conundrum, we determined the crystallographic structure of the GAF-PHY region from *SyB*-Cph1 as Pr. Surprisingly, this structure differs from canonical Phys by having a Pr *ZZZsyn,syn,anti* bilin configuration but shifted to the activated position in the binding pocket with consequent folding of the hairpin loop to α-helical, an architecture common for Pfr. Collectively, the PSM of *SyB*-Cph1 as Pr displayed a mix of dark-adapted and photoactivated features whose co-planar A-C pyrrole rings support a D-ring flip mechanism.

Most cellular organisms employ one or more photoreceptors to entrain their growth, development, and physiology to their ambient light environment. One major class in microorganisms and plants is the phytochromes (Phys), a diverse collection of dimeric chromoproteins that employ a covalently-bound bilin (or open chain tetrapyrrole) prosthetic group that can assume two conformers, a red light-absorbing Pr state and a metastable far-red light-absorbing Pfr state (1, 2). Through fast photointerconversion between Pr and Pfr and slower thermal reversion of Pfr back to Pr, Phys acts as photoswitches in detecting light fluence, duration, photoperiod, and the ratio of red and far-red light (3). Moreover, as thermal reversion is an enthalpic process, some Phys are also able to sense temperature through an accelerated loss of Pfr as temperatures rise (4, 5, 6). Normally, Pr is the dark-adapted, physiologically inactive state, while Pfr is the biologically active state but there are variants where Pfr is the dark-adapted state (bathyPhys) thus requiring far-red light for photoactivation, and unique families in algae and cyanobacteria named cyanobacteriochrome photoreceptors (CBCRs) that employ modified bilins to absorb wavelengths that collectively span the visible spectrum (1, 7, 8).

Phys are typically arranged as homodimers comprised of an N-terminal photo-sensing module (PSM) harboring the bilin followed by a C-terminal region that enables dimerization, and in the case of microbial Phys, often has one or more motifs for signal output (1, 2). The PSM houses sequential Per/ARNT/Sim (PAS), cGMP-specific phosphodiesterase/adenylyl cyclase/FhlA (GAF), and Phy-specific (PHY) domains, with a rare figure-of-eight knot strengthening the connection between the PAS and GAF domains (9). The bilin is buried within a GAF domain pocket, which through numerous chromophore/protein contacts provides the unique absorption and photochromicity of these photoreceptors.

While many details surrounding Pr→Pfr photoconversion remain unclear, a common step is the rotation of the bilin from a *ZZZssa* to *ZZEssa* configuration through *cis* to *trans* isomerization at the C15 = C16 methine bridge, which flips the D-pyrrole ring and induces the bilin to pivot within the GAF domain pocket (10, 11, 12, 13). This movement forces a hairpin (or tongue) loop extending from the PHY domain and contacting the GAF domain to refold from β-stranded to α-helical. Its subsequent rebinding to the GAF domain induces a ‘pull’ that reverberates into the PHY domain and beyond through twisting of central α-helical spines in the dimer, which in microbial Phys allosterically generates a signaling-competent Pfr state.

To better understand how Phys signals mechanistically, attention has been given to an assortment of microbial variants better suited to various spectroscopic and structural approaches. Included are mutants in canonical Phys that suppress thermal reversion (12), bathyPhys from proteobacteria that enables studies of Pfr without irradiation (14, 15, 16), and cyanobacterial Phys that retain normal photochromicity despite unique PSM arrangements (7). Examples of the latter include a plethora of CBCRs that utilize a single bilin-binding GAF domain without a hairpin for light detection, some of which retain photointerconversion while in a crystal lattice (17, 18, 19), the ‘knot-less’ Phy Cph2 from *Synechocystis* sp. PCC6803 (*Syn*) harboring a photointerconvertible GAF-GAF2 bidomain (20, 21), and a collection of PAS-less Phys related to *Syn*-Cph2 that retained most of the canonical architecture and Pr/Pfr photochemistry but lack the PAS domain and knot (22).

Of particular interest to us was the thermally stable PAS-less Phy (designated originally as *SyB*-Cph1 (22)) from the *Synechococcus* sp. JA-2-3B'a (2, 3, 4, 5, 6, 7, 8, 9, 10, 11, 12, 13) cyanobacterium isolated from a hot spring with characteristics accessible to NMR spectroscopy. Included were the discoveries that a fragment encompassing just the GAF domain remains red/far-red size light photoactive, thus placing it within the acceptable size range for 2D-NMR studies probing Pr and Pfr while also displaying the necessary stability for long-term NMR data collection (22). Consequently, through the NMR spectroscopic analyses of Pr and inherently heterogenous populations of Pfr generated by red light, we considered it possible to define Phy photointerconversion by comparing paired solution structures of *SyB*-Cph1(GAF) after labelling the apoprotein and its native bilin phycocyanobilin (PCB) with ^13^C and/or ^15^N (23, 24).

Toward this goal, Cornilescu *et al.* (24) described using 2D-NMR the first solution structure of the GAF domain from *SyB*-Cph1 as Pr and found that its architecture in the dark-adapted state largely resembles those determined for other microbial Phys by X-ray crystallography (*e.g*., (9, 14, 15, 25, 26, 27, 28)). Included were; (i) binding of PCB to the A-pyrrole ring *via* a thioether linkage to Cys138, (ii) positioning of the bilin in a *ZZZssa* configuration within a GAF domain pocket, (iii) proximity of the B- and C-ring propionate side chains to potential arginine anchors, and (iv) a tilt of the D-pyrrole ring stabilized by hydrogen bonds to neighboring amino acids. Follow-up 2D-NMR analysis of a light-driven mixture of Pr and Pfr then revealed the surprising possibility that *SyB*-Cph1(GAF) photoconversion might not involve rotation of the D ring as predicted but instead involve rotation of the A ring from one substantially out-of-plane relative to the B- and C rings as Pr to one more in the plane as Pfr (23). By contrast, subsequent studies of *SyB*-Cph1(GAF) by 2D magic angle spinning (MAS) NMR using samples where just the bilin was uniformly labeled with ^13^C and ^15^N came to the opposite conclusion that only the D ring rotates after red-light excitation, thus placing *SyB*-Cph1 photoconversion more in-line with canonical Phys (29). Unfortunately, both studies were complicated by inherent uncertainty in NMR-derived models that generate an ensemble of possibilities sometimes challenged by limited restraints, and the absence of the PHY domain and associated hairpin which might artifactually alter chromophore positioning and configuration (23, 29).

Here, we began to address this controversy by generating an X-ray crystallographic structure of *SyB*-Cph1 as Pr, in this case using the full PSM encompassing both the GAF and PHY domains and the connecting hairpin. This fragment assembled as a head-to-head dimer consistent with other bacterial Phys but included a unique GAF domain dimerization contact involving a 15-residue sequence previously defined as the Arg-Ile-Thr (RIT) motif that is conserved among PAS-less Phys (22), and relatively straight and parallel α-helical spines uncharacteristic of most microbial Phy structures. Surprisingly, our PSM structure also revealed a unique hybrid architecture that included a dark-adapted chromophore in a *ZZZssa* configuration typically seen for Pr but partially rotated to a “Pfr-like” position within the binding pocket along with an α-helical hairpin typically seen for Pfr. Taken together, our data provide support for a photoconversion mechanism implied by MAS-NMR (29) whereby the Pr state of *SyB*-Cph1 begins with co-planar A-C pyrrole rings, adds to Phy diversity, and suggests a natural mechanism for biasing the active conformation of a Phy.

## Results

### X-ray crystallographic structure of SyB-Cph1(GAF-PHY) as Pr

**Figure 1:**
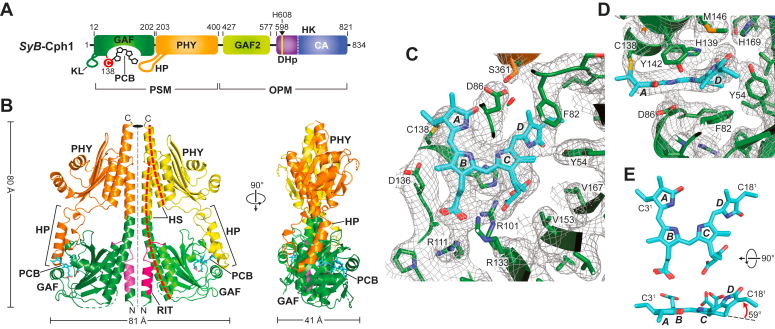
**3D model of the GAF-PHY fragment from *Synechococcus sp*. JA-2-3B'a** (2, 3, 4, 5, 6, 7, 8, 9, 10, 11, 12, 13) **(*SyB*) Cph1 assembled with PCB in the dark-adapted Pr state as determined by X-ray crystallography.***A*, diagram of full-length *SyB*-Cph1. The GAF, PHY, GAF2, and the DHp and CA domains within the HK bidomain are colored in *green*, *orange*, *light green*, *purple*, and *blue*, respectively. The orphan knot lasso (KL), hairpin loop (HP), and the predicted phosphoacceptor histidine (His608) in the DHp domain are indicated. C identifies Cys138 that covalently binds PCB *via* a thioether linkage. *B*, orthogonal views of the cartoon model for the GAF-PHY region. The GAF domain and PHY domain with its hairpin from each protomer are colored in *green* and *orange/yellow* respectively. PCB is shown in cyan sticks. The N-terminal α1(RIT) dimerization domains potentially unique to PAS-less Phys are colored in *magenta*. Helical spine, HS. Dimensions of the dimeric PSM are indicated. *C*, view of the PCB-binding pocket superposed with a 2mFo-DFc composite omit map contoured at 1σ (*grey mesh*) showing the position of PCB and neighboring amino acids. Arg101 and the C-ring propionate were modeled in two orientations. The nitrogen, oxygen, and sulfur atoms are in *blue*, *red*, and *yellow*, respectively. The A–D pyrrole rings are labeled. *D*, close-up views of PCB superposed with the omit map in panel (*C*) (*grey mesh*) showing the co-planar A–C pyrrole rings and the tilted D ring in a *ZZZssa* configuration, along with neighboring amino acids. *E*, side and top views of PCB shown in sticks illustrating the 59° tilt of the D ring. The C3^1^ carbon that participates in the thioether linkage with the apoprotein and the C18^1^ ethane carbon are labeled for reference.

Selenomethionine-enriched preparations of the GAF-PHY fragment from *SyB*-Cph1 (residues 1–421) assembled with its native chromophore—PCB—and bearing an in-frame C-terminal 6His tag were affinity purified by nickel-nitrilotriacetic acid (Ni-NTA) affinity chromatography followed by phenyl-Sepharose FPLC as described (9, 22) (Figs. 1*A* and S1*A*). It displayed repeatable red and far-red light photointerconversion with Pr and Pfr absorption maximum at 623 and 708 nm, respectively (Fig. S1*B*), consistent with harboring covalently-linked PCB (22). Using sparse matrix screening, conditions for the formation of blue tetragonal crystals were identified from dark-incubated samples (Fig. S1*C*). Absorbance spectra of these crystals confirmed a red-light absorption maximum consistent with Pr, with a small shoulder possibly reflecting residual Pfr (Fig. S1*D*).

A nearly complete diffraction dataset was collected from a single *SyB*-Cph1(GAF-PHY) crystal which enabled phasing by single wavelength anomalous dispersion (SAD). From these data, a 2.6-Å resolution model was built that included residues 12 to 117, 131 to 280, and 284 to 405 (PDB code 8W26). (See Table 1 for refinement statistics.) Electron density was absent for the N-terminal 11 residues and the sequence corresponding to the knot lasso (residues 118–130) that tethers the N-terminal extension from the PAS domain in canonical Phys (9), thus creating a flexible “orphan” loop in this PAS-less Phy.

**Table 1:** Data collection and refinement statistics for the GAF-PHY fragment of *SyB*-Cph1 (PDB ID 8W26)

Data collection and processing^a^	
Wavelength	0.9787
Resolution range	48.6–2.6 (2.63–2.6)^b^
Space group	P 4_3_ 2_1_ 2
Unit cell	102.846 × 102.846 × 130.516
Reflections	
Total	192,723 (6330)
Unique	40,848 (1381)
Multiplicity	4.7 (4.6)
Completeness (%)	99.72 (94.59)
Mean I/sigma(I)	12.44 (0.87)
Wilson B-factor	69.88
R_*merge*_	0.074 (1.50)
R_*meas*_	0.083 (1.70)
R_*pim*_	0.038 (0.78)
CC1/2	0.999 (0.48)
CC	1 (0.81)
Refinement statistics	
Reflections	
For refinement	22,009 (724)
For R-free	2201 (72)
R_*work*_	0.2004 (0.3358)
R_*free*_	0.2420 (0.4185)
Number of atoms	
Non-hydrogen	3419
Macromolecules	3147
Ligands	114
Solvent	158
Protein residues	378
RMSD	
Bonds	0.003
Angles	0.62
Ramachandran (%)	
Favored	98.12
Allowed	1.88
Outliers	0.00
Rotamer outliers (%)	0.90
Clashscore	7.84
B-factors	
Average	98.54
Macromolecules	99.57
Ligands	92.50
Solvent	82.32

a Collection and processing statistics were calculated in Phenix from the unmerged anomalous scalepack file generated by HKL-2000.

b Values in parentheses represent data from the highest resolution shell.

**Figure 2:**
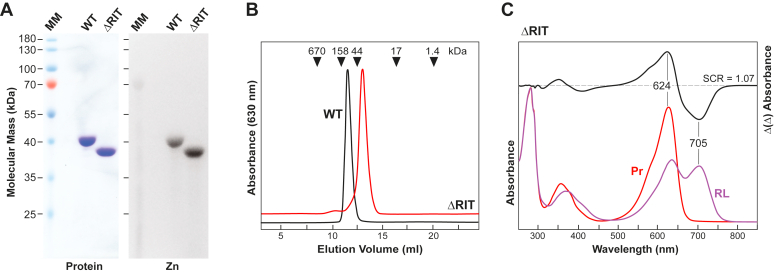
**Biochemical and spectroscopic characterizations of an *SyB*-Cph1(GAF-PHY) truncation examining the importance of the α1(RIT) helix in dimer assembly.***A*, SDS-PAGE analysis of the ΔRIT chromoprotein missing residues 2 to 29. The purified 6His-tagged samples were subjected to SDS-PAGE and the gels were either stained for protein with Coomassie *blue* or for the bound PCB by zinc-induced fluorescence. MM, molecular mass standards. *B*, SEC elution profile of the wild-type *SyB*-Cph1(GAF-PHY) fragment and the ΔRIT mutant. *Arrowheads* locate the positions of gel filtration mass standards used to calibrate the column. *C*, absorption and difference spectra of a 43-kDa ΔRIT mutant in panel (*B*) as Pr or after saturating irradiation with 630-nm *red light* to generate mostly Pfr (RL). The difference spectra are shown at 70% intensity. SCR, spectral change ratio (-ΔAbs_624nm_/ΔAbs_705nm_).

As shown in Figure 1*B*, *SyB*-Cph1(GAF-PHY) crystallized as a symmetric head-to-head dimer similar in fashion to many other Phys from bacteria (*e.g*., (14, 15, 26, 27)), and in agreement with size-exclusion chromatography (SEC) of the preparations showing that this fragment behaves as a dimer in solution ((22) see Fig. 2*B*). Superficial views of the dimeric model revealed a strong resemblance to those from corresponding regions in proteobacterial Phys (Fig. 1*B*), even though some other cyanobacterial Phy PSMs are monomeric in isolation as Pr and often crystallize as head-to-tail dimers (*e.g*., *Syn*-Cph1 and *Syn*-Cph2 (20, 25)). Similarities included a pair of parallel helical spines at the center of the dimer comprised of a long, central α-helix bounded on each end by a three-helix bundle that contacts the GAF domains on one end and the PHY domain on the other. The C-terminal α-helix exiting the PHY domains presumably continues into the downstream GAF2 and HK domains (see Fig. 1*A*), both of which were not included in the construction. While the helical spines in many bacterial Phys are bowed, this spine was relatively straight (Fig. 1*B*).

**Figure 3:**
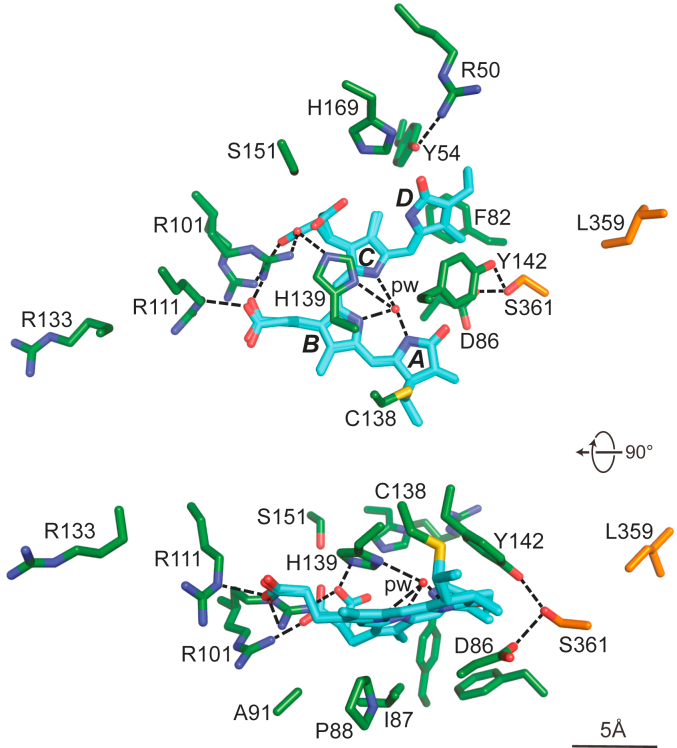
**Orthogonal stick views of the PCB-binding pocket of *SyB*-Cph1(GAF-PHY).** A–D pyrrole rings (*cyan*) and surrounding amino acids from the GAF and PHY domains (*green* and *orange*, respectively) are indicated. Arg101 and the C-ring propionate were modeled in two orientations. *Dashed lines* locate potential hydrogen bonds. The nitrogen, oxygen, and sulfur atoms are in *blue*, *red*, and *yellow*, respectively. pw, pyrrole water.

PCB was connected to the GAF domain by a thioether linkage between the C3^1^ carbon of the ethylidene side chain of PCB and the sulfur moiety of Cys138, and was buried within the GAF domain in the expected *ZZZssa* conformation for Pr. As with canonical Phys, the pyrroles of rings A-C along with an adjacent histidine imidazole group (His139) and the main chain carbonyl of Asp86 fixed a central water (designated pyrrole water (9)) (Fig. 3), which participates in proton release and uptake during photoconversion (1). Whereas the A-C pyrrole rings were mostly co-planar, the D ring was tilted by ∼59° relative to the C ring (Fig. 1, *D* and *E*).

### Eccentricities of the SyB-Cph1 architecture

**Figure 4:**
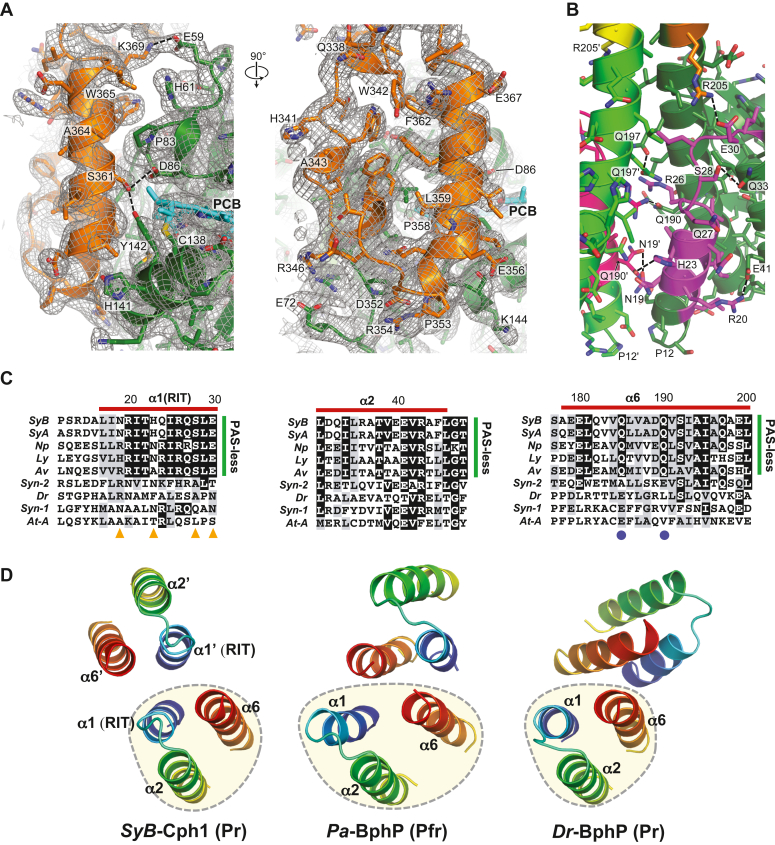
**Close-up views of the hairpin and α1(RIT) dimerization interface in the *SyB*-Cph1(GAF-PHY) model as Pr.***Dashed lines* in (*A*) and (*B*) indicate possible hydrogen bonds. *A*, orthogonal cartoon views of the α-helical hairpin region superposed with a 2mFo-Dfc composite omit map contoured at 1σ (*grey mesh*). *B*, close-up cartoon view of the N-terminal α1(RIT) helix and its interactions with the α2 and α6 helicies in the GAF domain. *C*, amino acid sequence homology surrounding the α1(RIT) helix and its companion interaction sequences in the GAF domain α2 and α6-helicies of its protomer. *Red lines* demarcate the helices. *Green lines* identify members of the PAS-less Phy family from cyanobacteria related in sequence to *SyB*-Cph1. *Blue circles* and *orange triangles* indicate possible hydrogen bond contacts between the GAF domain and α1(RIT) helix and the α6 helix, respectively. Amino acid sequences are from *SyB*-Cph1 (*SyB*) (YP_478662), *SyA*-Cph1 (*SyA*) (YP_476144), related PAS-less Cphs from *Nostoc punctiforme* PCC73102 (*Np*) (ZP_00111485), *Lyngbya* sp. PCC8106 (*Ly*) (ZP_01618934), and *Anabaena variabilis* (*Av*) (YP_324761), *Deinococcus radiodurans Dr*BphP (*Dr*) (lNP_285374), *Synechocystis* sp. PCC6803 Cph1 (*Syn*) (NP_442237) and Cph2 (BAA10536), and *Arabidopsis thaliana* PhyA (*At*) (AAC33219). The amino acid numbering is based on the *SyB*-Cph1 sequence. Full sequences of the GAF-PHY regions can be found in ref. (22). *D*, cartoon views of the six-helix bundle at the Phy dimer interface generated by the N-terminal region of the GAF domain. Shown are the bundles from *SyB*-Cph1, *Pa*-BphP as Pfr (PDB code 3IBR (14)), and *Deinococcus radiodurans* (*Dr*) BphP as Pr (PDB code 4Q0J (26)). The dashed yellow circles identify the contributing three helix bundle from one protomer. The α1 helix containing the RIT motif from *SyB*-Cph1 is labeled.

Despite these commonalities, a closer look revealed several surprises for the protomers in the *SyB*-Cph1(GAF-PHY) structure as Pr, including (i) an unexpected α-helical configuration of the hairpin, which is normally ascribed to the Pfr state (10, 14, 25, 26), (ii) a displacement of the bilin within the GAF domain, (iii) a unique arrangement of bilin/GAF domain pocket interactions, and (iv) an extended dimerization interface involving the RIT motif (Fig. 1, *B–D*). As shown in Figure 4*A*, the electron density map of the hairpin region easily identified one strand of the loop as α-helical, and like the Pfr state in other Phys (*e.g*., (10, 20, 26)), employed a serine (Ser361) in the hairpin to contact the GAF domain *via* Asp86 within the Asp-Ile-Pro (DIP) sequence close to the chromophore. While this serine is embedded with a consensus PRxSF motif in canonical Phys, it is within a variant PLISF motif in *SyB*-Cph1 whose arginine-to-isoleucine substitution might loosens the β-stranded hairpin contacts thus enabling folding to its “active” α-helical conformation.

**Figure 5:**
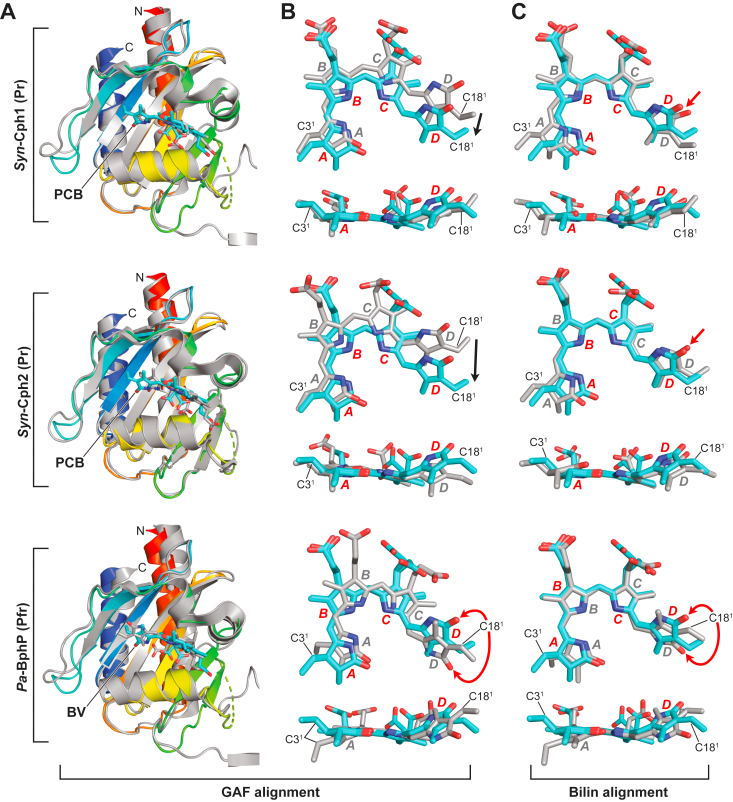
**The position of the bilin (PCB) in *SyB*-Cph1(GAF-PHY) assumes a unique position within the GAF domain pocket as compared to the bilin in other Phys.** Either Cα atoms of the crystallographic model of the GAF domain (*A* and *B*) or just the *B*- and *C*-pyrrole rings of the bilin from *SyB*-Cph1(GAF-PHY) were superposed with those of corresponding models as Pr from *Synechocystis* sp. PCC6803 Cph1 (*Syn*-Cph1) (PDB code 2VEA) and *Synechocystis* sp. PCC6803 Cph2 (*Syn*-Cph2) (PDB code 4BWI; (20)), or as Pfr from the bathyPhy *Pa*-BphP from *Pseudomonas aeruginosa* (PDB code 3C2W (14)). *SyB*-Cph1, *Syn*-Cph1, and *Syn*-Cph2 were assembled with PCB, whereas *Pa*-BphP was assembled with biliverdin (BV). *A*, superpositions of the cartoon models for GAF domains. The GAF domain of *SyB*-Cph1 is the colored in rainbow whereas the others are colored in *grey*. The positions of the bilins (*cyan*) are highlighted. N, N terminus. C, C-terminus. *B*, relative positions of bilins after GAF domain superposition. *C*, superpositions of the bilins aligned using the *B*- and *C*-pyrrole rings. In panels (*B*) and (*C*), the crystallographic models in sticks are colored in *cyan* and *grey*, respectively. The A-D pyrrole rings are identified. The C-ring propionate was modeled in two orientations. The C3^1^ carbon of PCB that forms the thioether linkage with the GAF domain cysteine, and the C18^1^ carbon are shown for reference. The nitrogen, oxygen, and sulfur atoms are in *blue*, *red*, and *yellow*, respectively. The *red arrows* in panels (*B*) and (*C*) highlight differences in the positions of the D-ring carbonyl in either the *Z* conformation (*SyB*-Cph1 *versus Syn*-Cph1 or *Syn*-Cph2) expected for Pr or the *E* conformation (*Pa*-BphP) expected for Pfr. The *black arrows* in panel (*B*) highlight partial sliding of the D ring to a ‘Pfr-like’ position in *SyB*-Cph1.

By a related event, the placement of PCB within the GAF-domain binding pocket unexpectedly also acquired a position resembling Pfr. As shown for paired models of Pr and Pfr (10, 12, 13), the bilin in canonical Phys pivots upon photoconversion within the binding pocket while remaining fixed at the A ring through its thioether linkage; a swivel that is likely caused by the amphipathic nature of the bilin moving to a more favorable hydrophobic/hydrophilic environment as the D ring rotates. For *SyB*-Cph1, we found a similar pivot despite PCB remaining as Pr. Superpositions based on the GAF domains comparing the bilin position of *SyB*-Cph1(GAF-PHY) to those in the Pr models of *Synechocystis* (*Syn*) Cph1 (25) and *Syn*-Cph2 (20) and the Pfr model of the bathyPhy from *Pseudomonas aeruginosa* (*Pa*-BphP) (14), showed that the GAF domains and the A-pyrrole ring positions aligned well despite photostate differences (Fig. 5). By contrast, the D ring was strongly displaced (2.1 Å for atom C16 of the D ring) as compared to the Pr models for *Syn*-Cph1 and *Syn*-Cph2 while being more congruent when compared to the Pfr position of *Pa*-BphP for not only the D ring but also for the B and C-rings. We speculate that this partial pivot is sufficient for generating an activated protein conformation with an α-helical hairpin, even though the bilin remains as Pr both conformationally and spectroscopically (Fig. 5, *B* and *C*).

Why the bilin was displaced in the *SyB*-Cph1 structure remains to be resolved, but the shift was accompanied by residue positions typically only seen for the Pfr state. For example, the C17 methyl carbon of *SyB*-Cph1 and the D-ring pyrrole nitrogen from the bathyPhy *Pa*-BphP occupied the same relative position within the binding pocket, which was adjacent to ‘activated’ positions for Asp86 and Tyr142 (Fig. 5). Additionally, several residues of the *SyB*-Cph1 bilin-binding pocket assume similar positions to those for *Pa*-BphP as Pfr (Fig. 3 and S2). These include migration of the D-ring away from His169, Pfr-like positioning of Tyr54 and Phe82, and hydrogen bonding between Ser361 in the hairpin PRxSF motif (PLISF) and Asp86 and Tyr142 of the GAF domain. However, because the C15 = C16 double bond remained in the *Z* configuration, the pyrrole nitrogen of the D-ring failed to hydrogen bond with the Asp86 carboxylate (Fig. 3).

Similar to cyanobacterial and plant Phys (1), *SyB*-Cph1 employs a conserved arginine—Arg101—to anchor the propionate groups but conversely uses Arg111 rather than the conserved knot-lasso residue Arg133 as the second anchor (Fig. 3). This residue switch is also seen in *Syn*-Cph2, which also lacks the PAS domain; in this case using Lys104 to bind the B-ring carboxylate (20). To accommodate this switch, both *SyB*-Cph1 and *Syn*-Cph2 extend the GAF domain β-sheet by one strand, which includes Arg111 and Lys104, respectively, as well as displace the lasso loop to block the propionate-binding potential of Arg133 (Fig. S2). In fact, *Syn*-Cph2 does not even contain an arginine at the position analogous to Arg133 but rather substitutes Thr124, indicating a loss of function.

To examine the relative importance of the Arg101, Arg111, and Arg133 proximal to the B- and C-ring propionates, we analyzed a collection of alanine substitutions. All three recombinant proteins like wild-type *SyB*-Cph1(GAF-PHY) mostly behaved as dimers by SEC (data not shown) and assembled with PCB to generate a Pr conformer with an absorbance maximum at ∼622 nm (Fig. S3*B*). Assessment of bilin occupancy by absorption spectra and by comparing protein levels *versus* zinc-induced bilin fluorescence implied that arginine mutants bound PCB less efficiently, suggesting that all three are important for the integrity of the binding pocket (Fig. S3*A*). By contrast, we found that while the Arg101-Ala mutant readily transitioned to Pfr upon red-light excitation, photoconversion of the Arg111-Ala and Arg133-Ala mutants was substantially blocked even after prolonged irradiations, suggesting that these two arginines are critical to the Pr→Pfr transition (Fig. S3*B*).

### Extended dimerization interface in the GAF domain

Initial phylogenetic analyses of PAS-less Phys by Ulijasz *et al.* (22) noted unique conservation for a 15-amino acid sequence proximal to the GAF domains, which they designated as the RIT motif based on inclusion of an invariant Arg-Ile-Thr sequence (Fig. 4*C*). Our model of *SyB*-Cph1 revealed that this region acquires an α-helical conformation that contributes to the dimerization interface of PAS-less Phys. As can be seen in Figures 1*B*, and 4, *B* and *D*, we found that this α1(RIT)-helix helps create a three-helix bundle with helix α2 and helix α6 within its protomer (previously designated as α1 and α5 (24)), which are connected by numerous hydrophobic interactions and several well-placed hydrophilic contacts (Fig. 4*C*). Although this interaction is not novel among Phys, the association of sister three-helix bundles in *SyB*-Cph1 forms a unique dimer interface not seen in Phys previously described in the PDB (Fig. 4*D*). Here, the sister α1(RIT)-helices formed contacts at the dimer interface, which excluded direct contacts between the α6-helices, while the opposite scenario is true for the other two major types of GAF domain interfaces found in head-to-head bacterial Phys represented by *Pa*-BphP (14) and *Dr*-BphP (26) (Fig. 4*D*).

An intriguing possibility was that the α1(RIT) helix as part of the three-helix bundle uniquely helps stabilize the dimeric PSM interactions of *SyB*-Cph1 without the PAS domain and its contributing figure-of-eight knot. Its absence in *Syn*-Cph1 and *Syn*-Cph2 might then explain why their PSMs are monomeric in solution but arranged likely artifactually as head-to-tail dimers *in crystallo* (20, 25). At least for the GAF-PHY fragment of *SyB*-Cph1 studied in isolation, we found by testing a truncation missing the unstructured N-terminus and all of the RIT motif (ΔRIT; residues 2–29 deleted) that this sequence does help in dimer assembly. While the ΔRIT truncation did not appreciably impact PCB binding nor Pr→Pfr photoconversion (Fig. 2, *A* and *C*), it did impair dimerization. The ΔRIT polypeptide behaved by SEC mostly as a monomer of 58 kDa *versus* the wild-type polypeptide that behaved as a dimer of 124 kDa (Fig. 2*B*).

### Comparisons of the crystallographic and NMR structures of the SyB-Cph1 GAF domain

**Figure 6:**
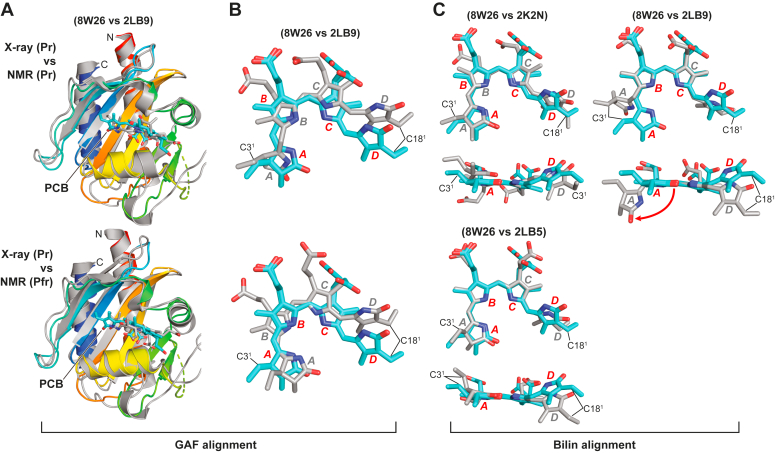
**Comparisons of the GAF domain models for *SyB*-Cph1 determined here for Pr by X-ray crystallography of a GAF-PHY fragment to that determined previously for Pr and Pfr by 2D-NMR using just the GAF domain.** The NMR models are based on the studies of Pr and Pfr by Ulijasz *et al.* (23) (PDB codes 2LB9 and 2LB5, respectively) and for Pr alone based on the studies of Cornilescu *et al.* (24) for Pr (PDB code 2K2N). All 2D-NMR structures were derived from the lowest energy models. Comparisons were conducted as described in Figure 5. *A*, superpositions of the cartoon models for GAF domains. The GAF domain from the X-ray crystallographic structure is colored in rainbow whereas the 2D-NMR structures are colored in *grey*. The positions of PCB (*cyan*) are highlighted. N, N terminus. C, C-terminus. *B*, relative positions of PCB after the GAF domains superposition highlighting the shift of the bilin within its binding pocket. *C*, superpositions of the bilins aligned using the *B*- and *C*-pyrrole rings. The out-of-plane orientation of the A ring in the 2LB9 NMR model of the GAF domain as Pr is highlighted by the *red arrow*. In panels (*B*) and (*C*), the crystallographic and NMR models are colored in *cyan* and *grey*, respectively. The C-ring propionate was modeled in two orientations. The A–D pyrrole rings are identified. The C3^1^ carbon of PCB that forms the thioether linkage with the GAF domain cysteine and the C18^1^ carbon are shown for reference. The nitrogen, oxygen, and sulfur atoms are in *blue*, *red*, and *yellow*, respectively.

As stated earlier, the initial impetus for solving the X-ray crystallographic structures of PCB and its binding pocket in *SyB*-Cph1 was to help resolve the conflict between opposing models of Pr→Pfr photoconversion determined by 2D-NMR and MAS-NMR, which proposed rotation of either the A- or D-pyrrole rings, respectively (23, 29). Here, we compared by superposition the crystallographic model for Pr determined here to the original 2D-NMR model of Pr determined for the GAF domain (PDB code 2K2N (24)), and to subsequent 2D-NMR models for the GAF domain that directly compared Pr and Pfr (PDB codes 2LB9 and 2LB5 (23)). Surprisingly, strongly different structural predictions emerged between the NMR and crystallographic models when assessing just the GAF domains, which also differed by oligomeric state (monomer *versus* dimer (Fig. 6)). While the overall folds were similar, excepting for the rearrangement of strands involving the lasso-loop and the presence of the α1(RIT) helix, superpositions of the α-carbon backbones revealed low congruity through whole model comparisons (rmsd ∼ 4.5 Å, 157 matching atoms), which improved when rejections of the conflicting positions were allowed (rmsd ∼1.3 Å, ∼136 matching atoms; Fig. 6). Additionally, PCB placements within the GAF domain pocket for all three NMR models were shifted relative the crystallographic model after superpositions based on the GAF domains, which themselves also varied depending on which 2D-NMR model was aligned (Fig. 6*B*).

When further comparing the configuration of PCB by itself after superposition of just the bilin either as Pr or Pfr, we found that the bilin in the original Pr model (PDB code 2K2N) by Cornilescu *et al.* (24) matched the crystallographic model reasonably well despite its altered placement in the binding pocket (Fig. 6*C*). Here, the A-pyrrole ring assumed a nearly co-planar orientation relative to the B- and C rings while the D ring was slightly out of plane like that in the crystallographic models seen here and in canonical Phys (9, 14, 15, 25, 26, 27, 28). However, when the PCB structures derived from the paired 2D-NMR models by Ulijasz *et al.* (23) defining both Pr and Pfr were compared (PDB codes 2LB9 and 2LB5, respectively), a strongly different PCB configuration of Pr was seen as compared to the crystallographic model (Fig. 6*C*). Although the positions of the B-D pyrrole rings superposed reasonably well, the A ring was considerably out-of-plane in the 2D-NMR structure, a position not found in the original 2D-NMR structure (24) nor in the crystallographic structure determined here (Fig. 1, *C* and *E*).

From further analysis of surrounding amino acids, we also found numerous positional discrepancies in the 2D-NMR *versus* crystallographic models of Pr, including placements of Tyr54, Phe82, Asp86, Arg101, Arg111, and Arg133; some favored interactions with PCB while others implied weaker interactions (Fig. S5). Such differences in the Pr positions of Tyr54 and Phe82 might be explained for the NMR model if the crystallographic structure reflected a subpopulation of particles in solution that acquired an “active” conformation. However, this does not seem to be the case as the Pfr structure determined by 2D-NMR also did not align with the crystallographic Pr structure except for having a similar configuration of the pyrrole rings A-C. Differences in the positioning of the lasso loop strands also led to large discrepancies in potential arginine-propionate contacts. As examples, Arg111 was calculated to be ∼10-Å further away from the B-ring propionate, while Arg133 was calculated to be ∼5-Å closer in the 2D-NMR *versus* crystallographic models.

Based on the crystal structure, a main conflict in the 2D-NMR model of Pr by Ulijasz *et al.* (23) was the proposed out-of-plane A ring (23); its position would clash with that of the carboxylate side chain of the DIP motif Glu86 (9). We speculate that the placement of Glu86 in the NMR structure erroneously migrated its side chain to an untenable position, likely due to the inability of 2D-NMR to track oxygen atoms. Regardless of the reason(s), the crystallographic data clearly favor a *ZZZssa* Pr model with only the D ring out-of-plane as seen for canonical Phys (1, 2) and that proposed by MAS-NMR analysis of *SyB*-Cph1 (29).

## Discussion

Since the first 3D structure of the PAS-GAF domains from *Dr*-BphP (9), an expanding collection of crystallographic models has been published from a bevy of Phys, thus revealing large variations in PSM architectures, including canonical PAS-GAF-PHY, bathyPhy, knot-less GAF-GAF2 bidomain, and CBCR subtypes (1, 2, 7). Here, we present a structure from the PAS-less Phy - *SyB*-Cph1 that potentially adds to this diversity. It crystallized with the chromophore in the Pr state, which, nonetheless, slid into its presumed ‘activated’ position in the binding pocket coincident with the hairpin also transitioning to its active, α-helical conformation. Additionally, a number of residues surrounding the bilin assumed Pfr-type rotamers. Surprisingly, despite these differences as well as additional amino acid repositionings/substitutions, it is interesting to note that the absorption spectra of *SyB*-Cph1 as Pr and Pfr are reasonably close to those of other cyanobacterial Phys that use a PCB chromophore ((20, 22, 25) Figs. 1*B* and 2).

Beyond these distinctions, it also appears that *SyB*-Cph1 further expands the structural diversity of the Phy superfamily for how it associates with head-to-head dimers through its GAF domain contacts. Here, the dimerization interface sported a compact arrangement between sister GAF domains involving the signature RIT motif preceding the GAF domain. A comparable interaction was not evident in *Syn*-Cph2 (20), which is also devoid of the PAS domain and whose PSM crystallized as a head-to-tail dimer, suggesting that the contacts in *SyB*-Cph1 are unique to the PAS-less Phy subfamily which shares a common domain architecture and sequence homology, especially within the GAF domain, hairpin, and RIT motif (22). An intriguing possibility is that this cyanobacterial PAS-less architecture evolved the α1(RIT) helix to confer strong thermal stability even without the PAS domain and its figure-of-eight knot contacts, thus maintaining Phy functionality in extreme environments such as hot springs (22) or aquatic mats subject to wide temperature extremes (*e.g*., *Lyngbya* and *Noctoc punctiforme*). Accordingly, we found that the N-terminal RIT motif helps stabilize the dimer; while the wild-type *SyB*-Cph1(GAF-PHY) fragment behaved as a dimer at moderate temperatures, a ΔRIT truncation was mostly monomeric.

Although the α-helical hairpin generally has been associated with the Pfr photostate (*e.g*., (10, 12, 13, 27)), we note that this configuration has also been recently reported for Pr crystals of the PSM from wild-type *Dr*-BphP and its Tyr263-Phe substitution altering the PRxSF motif (30). For the Tyr263-Phe mutant, a well-resolved “active” α-helical hairpin was seen even though the chromophore assumed the Pr configuration and an “inactive” β-stranded hairpin was present in solution as determined by small-angle X-ray scattering. For wild-type *SyB*-Cph1 that utilizes a PLxSF sequence instead, the arginine-to-isoleucine substitution might loosen the β-stranded hairpin contacts with the GAF domain *via* loss of the conserved Asp-Arg salt-bridge between the DIP and PRxSF motifs, thus resembling the *Dr*-BphP(Tyr263-Phe) mutation. Presumably, these substitutions could alter the structural equilibrium between “inactive” and “active” conformations of the PSM in the Pr state to allow the hybrid form that we see here for *SyB*-Cph1 by crystallography. And as Takala *et al.* (30) suggested, such changes could also be forced by crystallization conditions. In a related situation, the bathyPhy *Xanthomonas campestris* BphP, which is predominately Pfr in darkness, was initially crystallized as Pr due to an equilibrium between Pfr and Pr in the absence of light that allowed the trickling of Pfr into primarily Pr-state crystals (15). In a less likely supposition, *SyB*-Cph1 could represent a mechanistically distinct offshoot of Phys that starts as Pr in a hybrid state. However, there is insufficient mechanistic data to currently support such a hypothesis, nor is it clear how a Pr→Pfr transition in *SyB*-Cph1 would proceed as a D-ring flip might simply retain the α-helical hairpin.

As indicated earlier, the crystallographic 3D structure of *SyB*-Cph1(GAF-PHY) as Pr provided an opportunity to also test whether this Phy uses an A-ring *versus* D-ring flip during Pr→Pfr photoconversion as offered by contrasting 2D-NMR and MAS-NMR studies, respectively (23, 29). The main basis for the A-ring flip was provided by paired 2D-NMR structures from Ulijasz *et al.* (23), which proposed that the B-D rings are mostly co-planar for Pr and Pfr, while the A ring is substantially out of plane as Pr but more in plane as Pfr due to an ∼91° photostate-induced rotation. As shown in Figures 1, *A* and *D*, and 3, the crystallographic model of the *SyB*-Cph1(GAF-PHY) fragment as Pr does not support an out-of-plane A ring and is thus more in tune with the MAS-NMR studies. The bilin conformation as Pr provided by the crystallographic model also aligned well with those from a variety of other Phys which found an out-of-plane tilt for the D-pyrrole ring relative to the more co-planar A-C rings ((1) Fig. 1*E*).

There are a number of possibilities underpinning the disagreements in the crystallographic *versus* 2D-NMR models for *SyB*-Cph1. First and foremost could be the lack of sufficient 2D-NMR restraints to properly model the bilin and surrounding amino acids despite using a suite of NMR methods (residual dipole coupling and heteronuclear single-quantum coherence spectra) together with ^13^C and ^15^N labeling of both PCB and the apoprotein. This challenge to NMR approaches (both 2D-NMR and MAS-NMR) was also apparent when comparing the residues surrounding the chromophore, which for example placed Arg133, His169, and Lys52 as important PCB contacts although the crystal structure of Pr placed them considerably distant ((23, 24, 29); Fig. 3). Another possibility is that the presence of the PHY domain and its hairpin in the PSM crystal structure substantially influenced the positions of the bilin and surrounding amino acids in ways that were absent in the NMR experiments interrogating just the GAF domain. Indeed, the different impacts of the Arg101-Ala *versus* Arg133-Ala mutations affecting the B- and C-ring propionate contacts on Pr→Pfr photoconversion of the GAF domain alone *versus* the GAF-PHY fragment testify to this differential influence ((24); this report). Such a disparate impact might also explain why the structure of the α1(RIT) sequence was evident in the GAF-PHY dimeric structure but absent in the GAF domain only monomeric structure (this report; (23, 24)). Although unlikely, it remains also possible that the crystallographic structure of *SyB*-Cph1(GAF-PHY) only interrogated one of an ensemble of solution structures seen by 2D-NMR.

Whatever the reasons, our X-ray crystallographic data support a more canonical phototransformation mechanism for *SyB*-Cph1 as seen for the paired crystallographic models of canonical Phys (1, 2) and the MAS-NMR studies of *SyB*-Cph1 (29) whereby a flip of the D-pyrrole ring related to the planar A-C rings mostly likely underpins photoconversion of this PAS-less Phy from its dark-adapted to light-activated state. Moreover, given that both NMR methods misassigned several bilin/protein associations (*e.g.*, electrostatic contacts involving Arg133, His169, and Lys52) while separate 2D-NMR models of the GAF domain as Pr provided two different orientations of the A-pyrrole ring (23, 24, 29), NMR approaches might be inherently challenged in determining Phy structures alone, especially when attempting to structurally decipher the chromophore-binding pocket. Regardless, final confirmation of a likely *ZZZssa* to *ZZEssa* flip of PCB driven by a light-induced isomerization C15 = C16 methine bridge for *SyB*-Cph1 might ultimately require X-ray crystallographic resolution of the Pfr conformer.

## Experimental procedures

### Protein expression and purification

The coding region for amino acid residues 1 to 421 of the *SyB*-Cph1(GAF-PHY) apoprotein from *Synechococcus_sp*._JA-2-3B'a (2, 3, 4, 5, 6, 7, 8, 9, 10, 11, 12, 13) was cloned into a modified pBAD-MycHisC plasmid to bear an in-frame C-terminal 6His tag (SLHHHHHH), whose insertion position allowed expression control by the *AraBp* promoter (22). This plasmid was co-expressed in *Escherichia coli* BL21 AI cells along with a pL-PCB plasmid harboring the Ho1 and PcyA coding regions for PCB synthesis from *Synechocystis* sp. PCC6803 under the control of the *lac* operon (31). Cells at this and all subsequent steps were cultured at 37 °C overnight in 1 L of repression medium, collected by centrifugation, and resuspended in 6 L of M9 minimal medium supplemented with 600 mg each of L-lysine, L-threonine, L-phenylalanine, 300 mg each of L-leucine, L-isoleucine, L-valine, and L-selenomethionine, 15 mg of thiamine, 6 mg each of biotin, choline, folic acid, niacin, pantothenic acid, and pyrimidoxyl phosphate, 0.6 mg of riboflavin, and 100 mg of FeCl_2_. Cultures were grown at 37 °C to an OD_600_ of 0.9 and then cooled briefly on ice. One hundred mg of 5-aminolevulinic acid (ALA) was added and the incubation was continued at 37ºC for 1 h. Isopropyl B-D-2-thiogalactopyranoside (IPTG) was then added to a final concentration of 1 mM to induce PCB synthesis. After an additional h, L-arabinose was added to 2 g/L to induce apoprotein synthesis and growth continued overnight at 20 °C.

Under green safelights, cells were collected *via* centrifugation and resuspended in 25 ml of lysis buffer (50 mM Tris-HCl (pH 8.0), 300 mM NaCl, 1 mM 2-mercaptoethanol, 1 tablet Roche complete (EDTA-free) protease, 10% glycerol, 20 mM imidazole, 0.05% Tween 20, and 1 mM phenylmethylsulfonyl fluoride). Harvested cells were frozen in liquid nitrogen and stored at −80 °C. The cells were thawed and lysed by repeated sonication on wet ice for 10 min, and the lysate was then clarified by centrifugation at 30,000*g* for 30 min. The supernatant was passed through a 15-mL column of Ni-NTA beads (Qiagen Sciences) which were pre-equilibrated with the lysis buffer. Captured *SyB*-Cph1(GAF-PHY) was eluted with lysis buffer supplemented with imidazole to 400 mM. *SyB*-Cph1-containing fractions were further purified *via* hydrophobic interaction FPLC using a 17-mL HP butyl-Sepharose column (GE Healthcare) in combination with a 300 to 0 mM ammonium sulfate gradient in 30 mM Tris-HCl (pH 8.0). Fractions containing *SyB*-Cph1(GAF-PHY) identified by UV-vis spectroscopy were pooled, concentrated, and passed through a 24-mL Superdex 200 column equilibrated with 10 mM HEPES-NaOH (pH 8.5) and 100 mM NaCl storage solution. The sample was concentrated to 17 mg/ml and flash frozen as 30 μl pellets in liquid nitrogen and stored at −80 °C.

### Crystallization conditions

Optimal crystallization conditions for selenomethionine-labeled *SyB*-Cph1(GAF-PHY) as Pr were identified in darkness by a Hampton Index screen set up with 14.5 mg/ml protein at 4 °C and 20 °C. Crystals of protein (at 17 mg/ml) were obtained with 11 to 17% (w/v) tacsimate (pH 7.0), 2% (w/v) PEG 3350, 2% (v/v) DMSO, and 100 mM MOPS (pH 7.0). Crystals were prepared for X-ray crystallography with a mother liquor solution of 18% (w/v) tacsimate, 100 mM MOPS (pH 7.0), 2% (w/v) PEG 3350, 2% (v/v) DMSO, and 23% (v/v) ethylene glycol.

### X-ray diffraction data collection and structural determination

X-ray diffraction data for selenomethionine-labeled protein were collected at 100 K at the Life Sciences Collaborative Access Team beamline (Argonne National Laboratories Advanced Photon Source). The data were indexed, integrated, and scaled using HKL2000 (32). Initial single-wavelength anomalous diffraction phasing was performed using Phenix-AutoSol (33). Subsequent GAF-PHY model building was executed manually using Coot (34), and refined with Phenix.refine (35). Refinement in Phenix included translation/libration/screw (TLS) group optimization, optimization of stereochemical and atomic displacement parameter weights, and secondary structure restraints. Structure validation was conducted using Molprobity (36). Superpositions and illustrations were prepared with the PyMol Molecular Graphics System (http://www.pymol.org/). Data collection, processing, and refinement statistics are shown in Table 1.

### Biochemical analysis of wild-type and mutant *SyB*-Cph1

The 6His-tagged coding region for *SyB*-Cph1(GAF-PHY) present in the pBAD-MycHisC plasmid (22) was modified by Gibson assembly (37) to harbor specific arginine to alanine codon substitutions or the ΔRIT truncation, which were verified as correct by DNA sequence analysis of full coding regions. *E*. *coli* BL21 AI cells containing the constructions were grown as above without supplements, and induced overnight for *SyB*-Cph1 expression with ALA, IPTG, and arabinose at 16 °C. The cultures were lysed by sonication in 30 mM Tris-HCl (pH 7.0), 300 mM NaCl, 1 mM 2-mercaptoethanol, 1 mM phenylmethylsulfonyl fluoride, 10% glycerol, 20 mM imidazole, and 0.05% (v/v) Tween 20, and incubated further for 1 h at ice temperatures. The lysate was clarified at 16,000*g* for 30 min, purified by Ni-NTA affinity chromatography using the wash buffer containing 300 mM imidazole for elution, followed by hydrophobic interaction FPLC using a butyl-HP column (GE Healthcare) combined with a 100 or 50 mM to 0 mM ammonium sulfate gradient in 30 mM Tris-HCl (pH 8.0) depending on the mutant. The *SyB*-Cph1 containing fractions, identified by UV-vis absorption, were exchanged into 30 mM Tris-HCl (pH 8.0) by SEC using a 300-mL Superdex 200 column equilibrated in the same buffer.

Sample purity was assessed by SDS-PAGE followed by staining of the gels for protein with Coomassie blue and detection of the bound bilin by zinc-induced fluorescence (22). UV-visible spectroscopy was conducted in triplicate at 22 °C using a Cary60 spectrophotometer (Agilent). Pr spectra were collected from dark-adapted samples whereas Pfr spectra were collected after a 10-min irradiation with a 630-nm LED at a fluence rate of 13.5 μmol m^−2^ sec^−1^ to saturate photoconversion (13). Absorbance spectra of *SyB*-Cph1(GAF-PHY) crystals were recorded using an Ocean Optics USB4000 spectrophotometer at the Brookhaven National Laboratory. SEC of Pr was accomplished in darkness with samples dissolved in 30 mM Tris-HCl (pH 8.0) using a Superdex 200 Increase 10/300 Gl column coupled to an AKTA FPLC (GE) (flow rate of 0.5 ml min^−1^) in combination with gel filtration mass standards (BioRad) for molecular mass calculations.

## Data availability

The atomic model of *SyB*-Cph1(GAF-PHY) as Pr has been deposited in the RCSB Protein Data Bank (http://www.rcsb.org) under accession code 8W26. Models for *Synechocystis* sp. PCC6803 Cph1, *Synechocystis* sp. PCC6803 Cph2, *P. aeruginosa* BphP, and *Deinococcus radiodurans* BphP were obtained from the RCBS Protein Data Bank under accession codes 2VEA, 4BW1, 3IBR, and 4Q0J, respectively. Atomic coordinates and structural constraints for the 2D-NMR models of *SyB*-Cph1(GAF) as Pr (PDB codes 2K2N and 2LB9) and Pfr (PDB code 2LB5) are available in RCSB Protein Data Bank.

## Supporting information

This article contains supporting information.

## Conflict of interest

The authors declare that they have no known competing financial interests or personal relationships that could have appeared to influence the work reported in this paper.
