# Supplementary material for: Crystal structure of the photosensory module from a PAS-less cyanobacterial phytochrome as Pr shows a mix of dark-adapted and photoactivated features
Source: J Biol Chem. 2024 May 14;300(7):107369. doi: 10.1016/j.jbc.2024.107369 (PMC11264168; doi:10.1016/j.jbc.2024.107369)
Supplement: Supplemental Figures S1–S4 [file mmc1.pdf]

Supporting Information

Crystal Structure of the Photosensory Module from a PAS-Less Cyanobacterial Phytochrome as Pr Shows a Mix of Dark-Adapted and Photoactivated Features

E. Sethe Burgie, Alayna J. Mickles, Fang Luo, Mitchell D. Miller, and Richard D. Vierstra

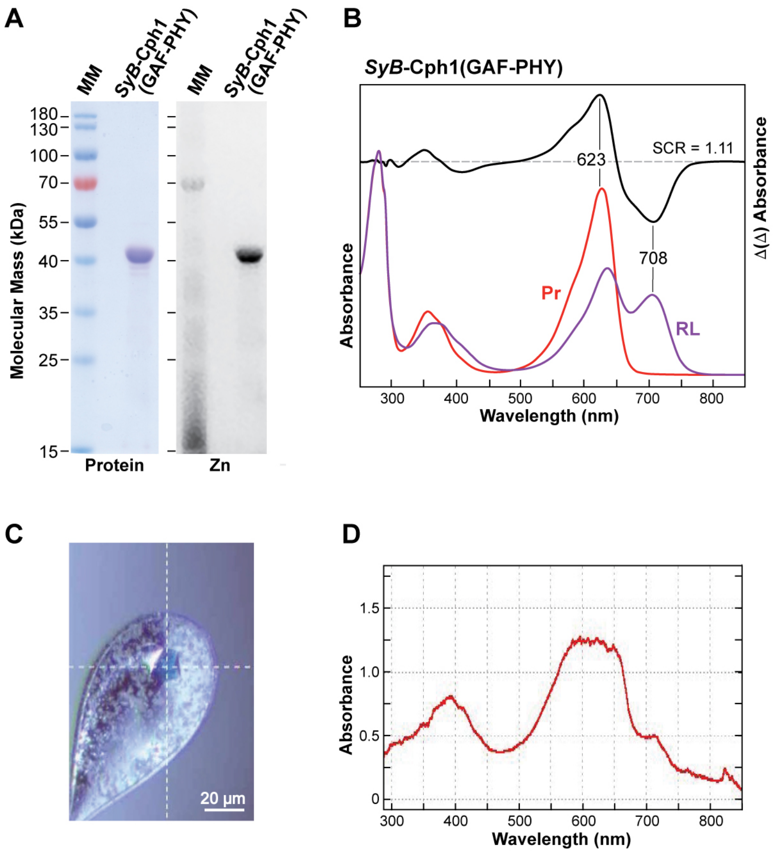

Supplemental Figure 1. Biochemical and spectroscopic characterizations of the *SyB*-Cph1(GAF-PHY) fragment assembled with PCB. ***A***, SDS-PAGE analysis of the purified chromoprotein. The samples were subjected to SDS-PAGE and the gels either stained for protein with Coomassie blue or for the bound PCB by zinc-induced fluorescence. MM, molecular mass standards. ***B***, Absorption and difference spectra of the preparations as Pr or after saturating irradiation with 630-nm red light to generate mostly Pfr (RL). The difference spectrum is shown at 70% intensity. SCR, spectral change ratio ($-\Delta Abs_{623nm}/\Delta Abs_{708nm}$). ***C***, Photograph of a *SyB*-Cph1(GF-PHY) crystal before X-ray crystallography. ***D***, Absorption spectrum of the crystal in panel (*C*) showing an absorbance maximum in the red region of the visible spectrum as expected for the Pr state.

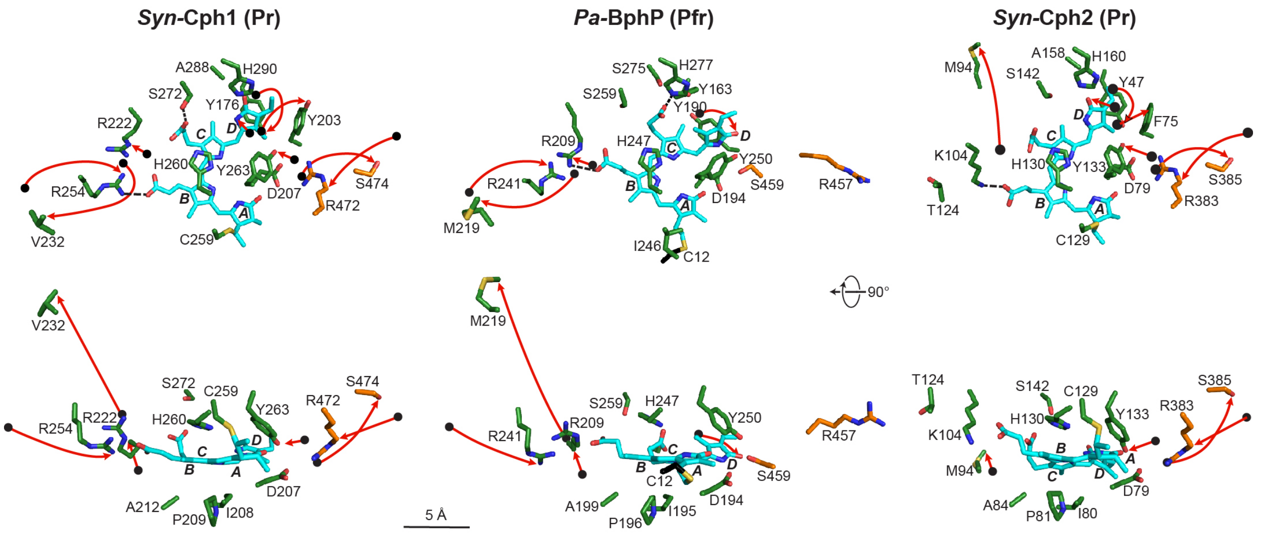

Supplemental Figure 2. Orthogonal cartoon view comparisons of the bilin-binding pocket determined by X-ray crystallography for the canonical Phy *Syn*-Cph1 as Pr from *Synechocystis* sp. PCC6803 (PDB code 2VEA (25)), the GAF-GAF-bidomain of *Syn*-Cph2 as Pr from *Synechocystis* sp. PCC6803 (PDB code 4BW1 (20)), and the bathyPhy from *Pseudomonas aeruginosa Pa*-BphP as Pfr (PDB code 3C2W (14)) relative to that of *SyB*-Cph1. *SyB*-Cph1, *Syn*-Cph1, and *Syn*-Cph2 were assembled with PCB, whereas *Pa*-BphP was assembled with biliverdin. The red arrows identify identical or analogous atoms from key amino acids that had markedly different positions relative to those in *SyB*-Cph1 (indicated by the black circles). The A-D pyrrole rings are indicated. The nitrogen, oxygen, and sulfur atoms are in blue, red, and yellow, respectively.

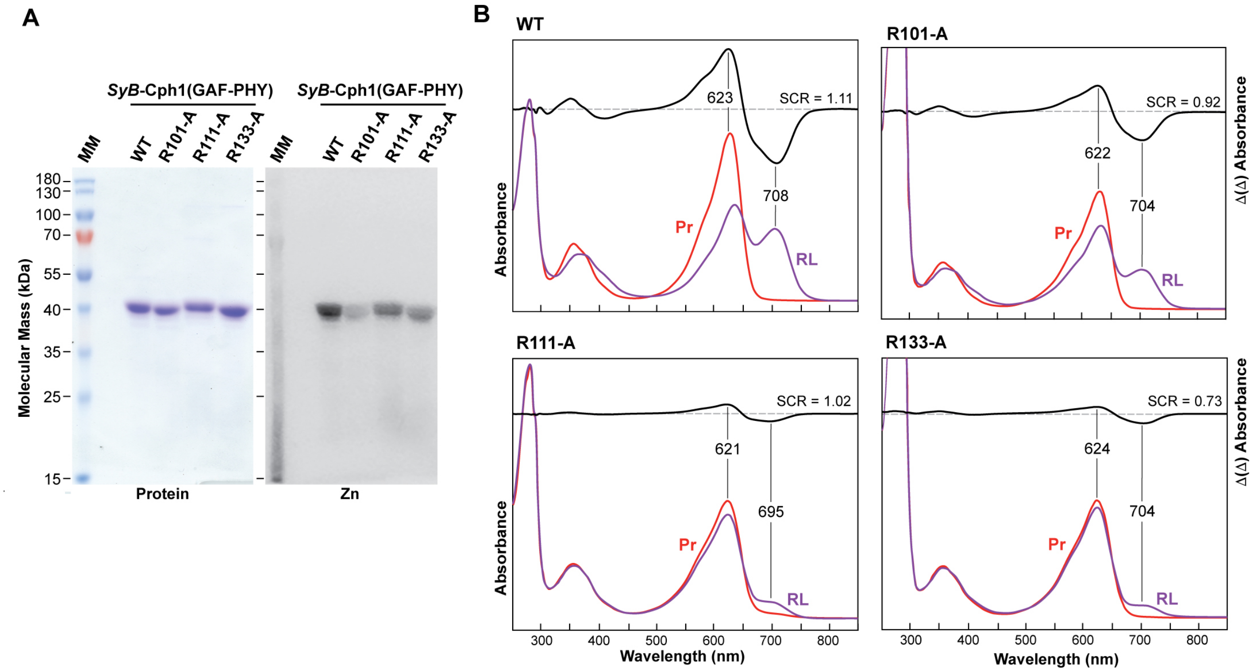

Supplemental Figure 3. Biochemical and spectroscopic characterization of *SyB*-Cph1(GAF-PHY) mutants examining the importance of several arginines near the B- and C-ring propionate groups. ***A***, SDS-PAGE analysis of the R101-A, R111-A, and R133-A substitutions. The purified 6His-tagged samples were subjected to SDS-PAGE and the gels were either stained for protein with Coomassie blue or for the bound PCB by zinc-induced fluorescence. MM, molecular mass standards. ***B***, Absorption and difference spectra of the preparations as Pr or after saturating irradiation with 630-nm red light in an attempt to generate Pfr (RL). The difference spectra are shown at 70% intensity. SCR, spectral change ratio ($-\Delta Abs_{623nm}/\Delta Abs_{708nm}$). The spectra for WT *SyB*-Cph1(GAF-PHY) was reused from Figure S1 to allow for direct comparison.

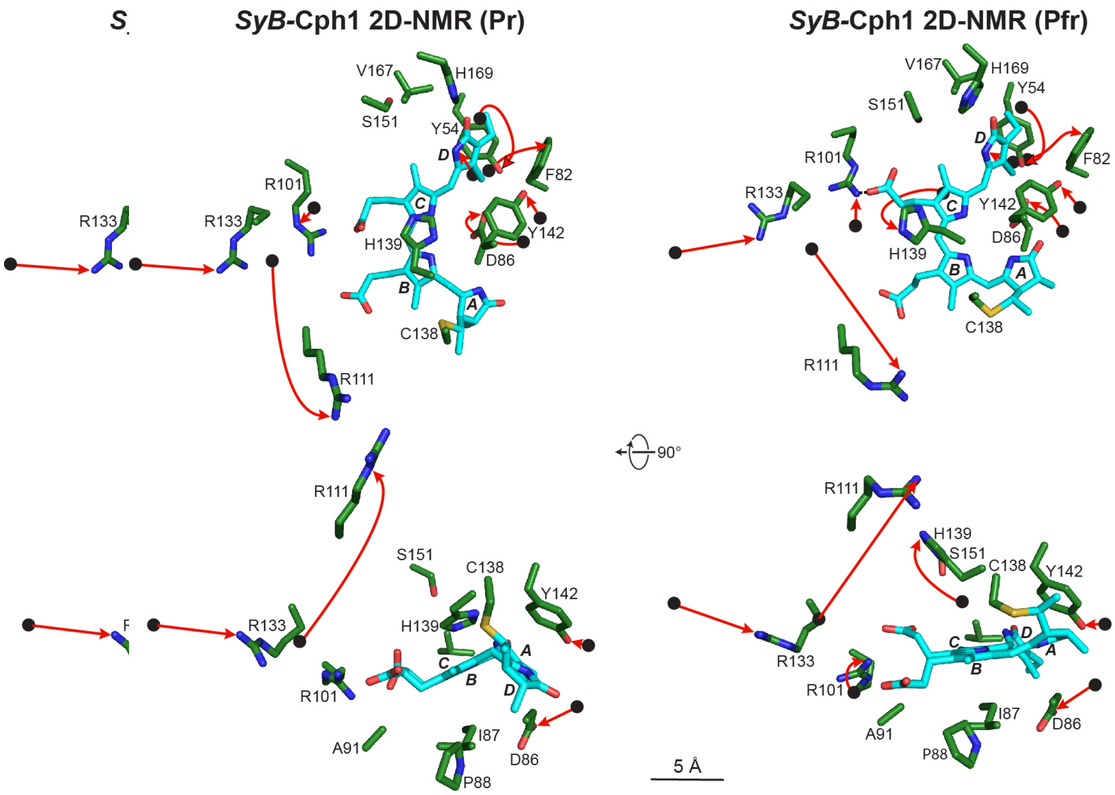

Supplemental Figure 4. Orthogonal cartoon view comparisons of the PCB-binding pocket of the *SyB*-Cph1(GAF) fragment determined previously by 2D-NMR as Pr and Pfr (PDB codes 2LB9 and 2LB5, respectively (23)) highlighting how surrounding amino acids assumed different positions when compared to the X-ray crystallographic structure of *SyB*-Cph1(GAF-PHY) as Pr. The 2D-NMR structures shown were derived from the lowest energy models. The red arrows highlight identifying atoms from key amino acids that had markedly different positions in the 2D-NMR models as compared to the those in the crystallographic model (positions indicated by the black circles). The A-D pyrrole rings are indicated. The nitrogen, oxygen, and sulfur atoms are in blue, red, and yellow, respectively.
